# Novel preparation of highly photocatalytically active copper chromite nanostructured material via a simple hydrothermal route

**DOI:** 10.1371/journal.pone.0158549

**Published:** 2017-06-05

**Authors:** Farshad Beshkar, Sahar Zinatloo-Ajabshir, Samira Bagheri, Masoud Salavati-Niasari

**Affiliations:** 1 Institute of Nano Science and Nano Technology, University of Kashan, Kashan, I. R. Iran; 2 Nanotechnology and Catalysis Research Centre (NANOCAT), IPS Building, University of Malaya, Kuala Lumpur, Malaysia; Brandeis University, UNITED STATES

## Abstract

Highly photocatalytically active copper chromite nanostructured material were prepared via a novel simple hydrothermal reaction between [Cu(en)_2_(H_2_O)_2_]Cl_2_ and [Cr(en)_3_]Cl_3_.3H_2_O at low temperature, without adding any pH regulator or external capping agent. The as-synthesized nanostructured copper chromite was analyzed by transmission electron microscopy (TEM), UV–vis diffuse reflectance spectroscopy, energy dispersive X-ray microanalysis (EDX), scanning electron microscopy (SEM), X-ray diffraction (XRD) and Fourier transform infrared (FT-IR) spectroscopy. Results of the morphological investigation of the as-synthesized products illustrate that the shape and size of the copper chromite depended on the surfactant sort, reaction duration and temperature. Moreover, the photocatalytic behavior of as-obtained copper chromite was evaluated by photodegradation of acid blue 92 (anionic dye) as water pollutant.

## Introduction

The preparation of nanosized materials has been the focus of recent scientific and technological investigation owing to their noteworthy and interesting characteristics [1–5]. Among these nanostructured materials, copper chromite (CuCr_2_O_4_) has attracted tremendous and considerable attention owing to its fascinating characteristics, and its excellent commercial applications in solid propellants, propulsion of rocket, chemical reactions, propellant combustion and photocatalytic H_2_ production [6–12]. A number of ways for the preparation of copper chromite have been introduced, such as hydrothermal, solid state reaction, nanocasting and thermal decomposition [12–16]. Since shape and particle size have key and substantial impact on the properties and final applications of the nanostructured materials, different ways have been exploring for size and shape controlled synthesis of nanostructured materials [3, 17–18]. Of the different routes of preparation of copper chromite, the hydrothermal way is well-known as a simple, cost-effective and reliable process to control the shape and size of nanostructured copper chromite [7, 19].

Here, we report a novel simple hydrothermal procedure to synthesize the nanostructured copper chromite utilizing [Cu(en)_2_(H_2_O)_2_]Cl_2_ and [Cr(en)_3_]Cl_3_.3H_2_O at low temperature. In this way, separated ethylenediamine (en) from [Cu(en)_2_(H_2_O)_2_]Cl_2_ and [Cr(en)_3_]Cl_3_.3H_2_O in the system has been utilized as both pH regulator and capping agent. In some cases, the pH regulator and capping agent as one of the starting materials has been added in the reaction system [14, 20]. To the best of our knowledge, it is the first time that [Cu(en)_2_(H_2_O)_2_]Cl_2_ and [Cr(en)_3_]Cl_3_.3H_2_O are applied for the hydrothermal preparation of nanostructured copper chromite and the influence of certain preparation factors on the shape and particle size of the copper chromite through a new facile hydrothermal procedure are examined.

## Experimental section

### Materials and characterization

All the chemicals applied for the synthesis of nanostructured copper chromite including CrCl_3_.6H_2_O, Cetyltrimethylammonium bromide (CTAB), CuCl_2_.2H_2_O, sodium dodecyl sulphate (SDS), zinc granule, ethylenediamine (en), HCl, polyvinylpyrrolidone (PVP-25000) and methanol were purchased from Merck Company and were applied as received. Morphological characteristics of the copper chromite samples were studied by a Hitachi S-4160 field emission scanning electron microscope (FESEM). The energy dispersive spectrometry (EDS) analysis was investigated by Tescan mira3 microscope. Fourier transform infrared spectra of the as-synthesized samples were obtained applying KBr pellets on an FT-IR spectrometer (Magna-IR, 550 Nicolet) in the 400–4000 cm^-1^ range. TEM micrographs of as-prepared nanostructured copper chromite were obtained on a JEM-2100 with an accelerating voltage of 200 kV equipped with a high resolution CCD Camera. Powder X-ray diffraction (XRD) patterns of as-synthesized products were collected from a Philips diffractometer applying X’PertPro and the monochromatized Cu Ka radiation (l = 1.54 Å). The UV-vis diffuse reflectance spectra of the as-produced nanostructured copper chromite were obtained on a UV-vis spectrophotometer (Shimadzu, UV-2550, Japan).

### Preparation of copper source

For synthesizing of the copper source, [Cu(en)_2_(H_2_O)_2_]Cl_2_, a stoichiometric amount of en (4 mol) was added drop-wise to a CuCl_2_.2H_2_O solution (2 mol in 100 ml of distilled water). The copper source (blue precipitate) was obtained after stirring the mixture for 1 h (at 60°C), separating by filtering, washing and air-drying.

### Preparation of chromium source

For synthesizing of the chromium source, [Cr(en)_3_]Cl_3_.3H_2_O, 10 ml of en was added drop-wise to the mixture (1 g of zinc granules was added to a CrCl_3_.6H_2_O solution (5.32 g of CrCl_3_.6H_2_O and 1 g of zinc granules in 20 ml of methanol). The chromium source (yellow precipitate) was obtained after refluxing the mixture for 1.5 h (at 60°C), collecting by filtering (removing the granules of zinc), washing (with 10% solution of en in methanol and then with ether) and air-drying.

### Preparation of copper chromite micro/nanostructures

**Fig 1:**
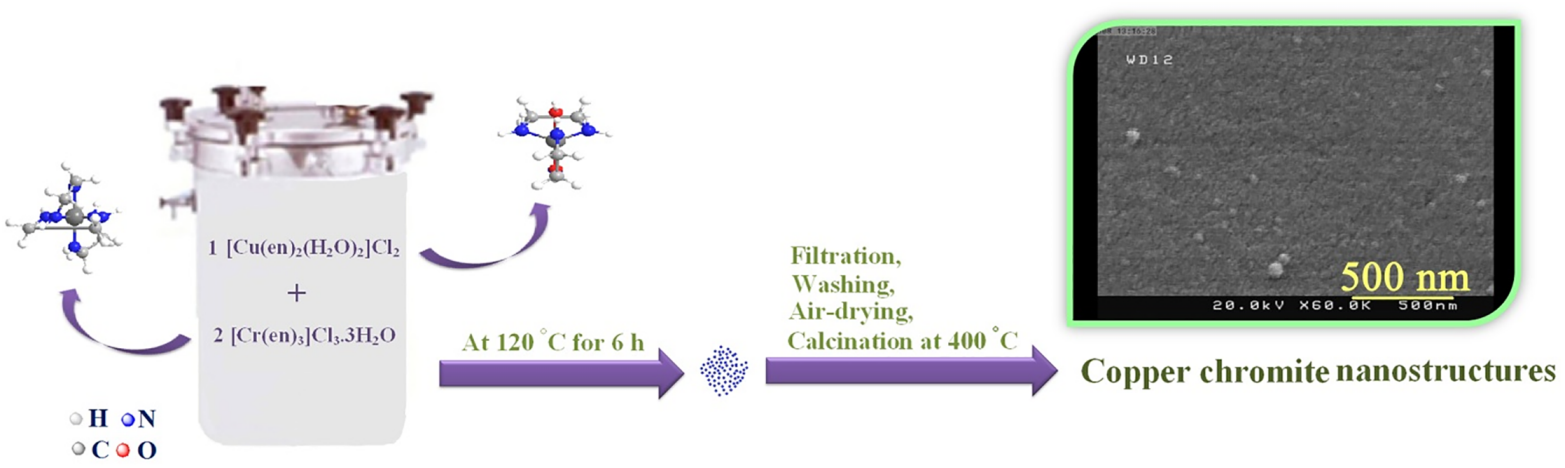
Schematic diagram of the preparation of the copper chromite nanostructures.

Copper chromite micro/nanostructures were prepared by a novel simple hydrothermal way. To synthesize copper chromite, in a typical experiment, 0.1 g of [Cu(en)_2_(H_2_O)_2_]Cl_2_ and 0.27 g of [Cr(en)_3_]Cl_3_.3H_2_O with a Cu:Cr molar ratio of 1:2, were dissolved in 20 ml distilled water separately. Chromium source solution was added drop-wise to the copper source solution under magnetic stirring. After 15 min stirring the resultant mixed solution was sealed in a 200 mL Teflon-lined stainless steel autoclave and maintained at 120°C for 6 h. The final precipitate was filtered, washed out with ethanol and distilled water for three times, air-dried and calcined at 400°C for 3h (sample no. 3). Schematic diagram of the preparation of copper chromite nanostructures is demonstrated in Fig 1. For examining the effect of the surfactant sort, a certain amount of the surfactant was dissolved in 5 ml distilled water and added after mixing chromium source and copper source solutions. The influence of the surfactant type, reaction duration and temperature on the shape and size of the copper chromite were also examined (Table 1).

**Table 1:** The synthesis conditions of the copper chromite micro/nanostructures.

Sample No.	Reaction temperature (°C)	Reaction duration (h)	Surfactant type	Calcination temperature (°C)	Figure of SEM images
1	120	6	-	600	-
2	120	6	-	500	-
3	120	6	-	400	4a
4	150	6	-	400	4b
5	180	6	-	400	4c
6	120	12	-	400	4d
7	120	18	-	400	4e
8	120	6	CTAB	400	4f
9	120	6	PVP	400	4g
10	120	6	SDS	400	4h
^a^11	120	6	-	400	4i

^a^ Blank test

### Photocatalytic test

The photocatalytic behavior of as-synthesized copper chromite nanostructures was examined by applying acid blue 92 (anionic dye) solution. The solution containing 0.001 g of the acid blue 92 and 0.04 g of the as-obtained copper chromite in the quartz reactor was applied to perform the photocatalytic test. After aerating for 30 min, the mixture was subjected to the irradiation of the visible light from the 400 W Osram lamps. The acid blue 92 photodegradation percentage was estimated as follow:

$$D.P.(t)=\frac{A_{0}-A_{t}}{A_{0}}\times 100$$ (1)

where A_t_ and A_0_ are the obtained absorbance quantity of the acid blue 92 solution at t and 0 min by a UV–vis spectrometer, respectively.

## Results and discussion

**Fig 2:**
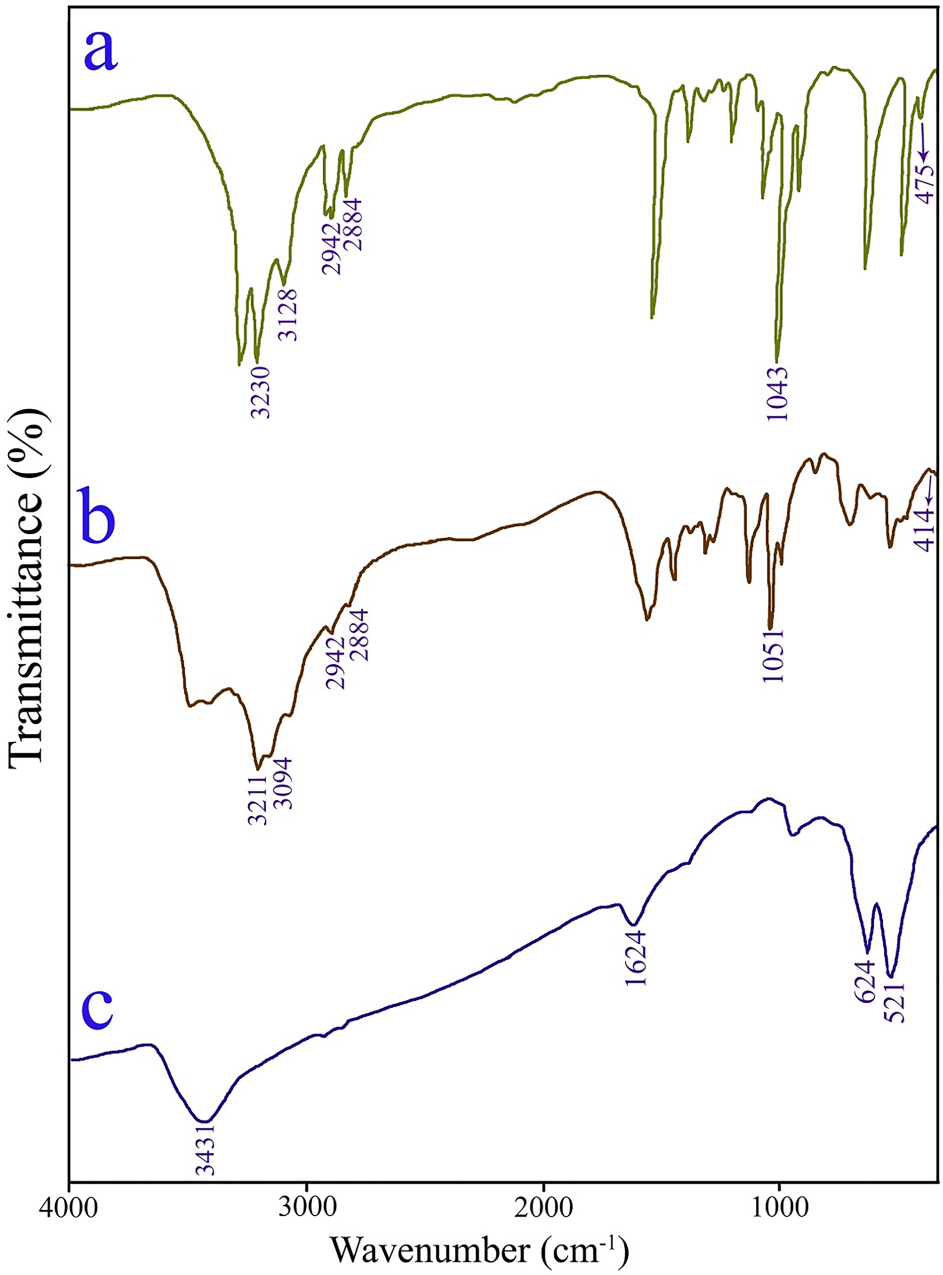
FT-IR spectra of copper source (a), chromium source (b) and copper chromite nanostructures (sample no. 3) (c).

In order to determine the formation of the chromium source, copper source and copper chromite, FT-IR analysis was carried out. Fig 2A–2C illustrates the infrared spectra of the copper source, chromium source and as-obtained copper chromite nanostructures (sample no. 3), respectively. In the FT-IR spectrum of the copper source (Fig 2A), the bands located at 3230 and 3128 cm^-1^ are attributable to the stretching vibration of N-H of the en [21]. Two bands seen at 2942 and 2884 cm^-1^ are corresponding to the symmetry stretching and asymmetry stretching mode of the CH_2_ groups of the en, respectively. The band located at 1043 cm^-1^ is attributable to the C–N stretching vibration. The band at 475 cm^-1^ is corresponding to *ν*(Cu–N) vibration [22]. FT-IR spectrum of the chromium source is seen in Fig 2B. The peaks located at 3211 and 3094 cm^-1^ in Fig 2B are attributable to the stretching vibration of N-H [21] and the bands centered at 2942 and 2884 cm^-1^ are ascribed to the symmetry stretching and asymmetry stretching mode of the CH_2_ groups of the en. The band observed at 1051 cm^-1^ is corresponding to the C–N stretching vibration (Fig 2B). Furthermore, the presence of *ν*(Cr–N) vibration at 414 cm^-1^ is seen [23]. In the case copper chromite nanostructures (sample no. 3), the peaks observed at 3431 and 1624 cm^–1^ are ascribed to the *v*(OH) stretching and bending vibration of the surface adsorbed water molecules [4]. The characteristic bands of the copper chromite centered at 624 and 521 cm^-1^ [24] (Fig 2C).

**Fig 3:**
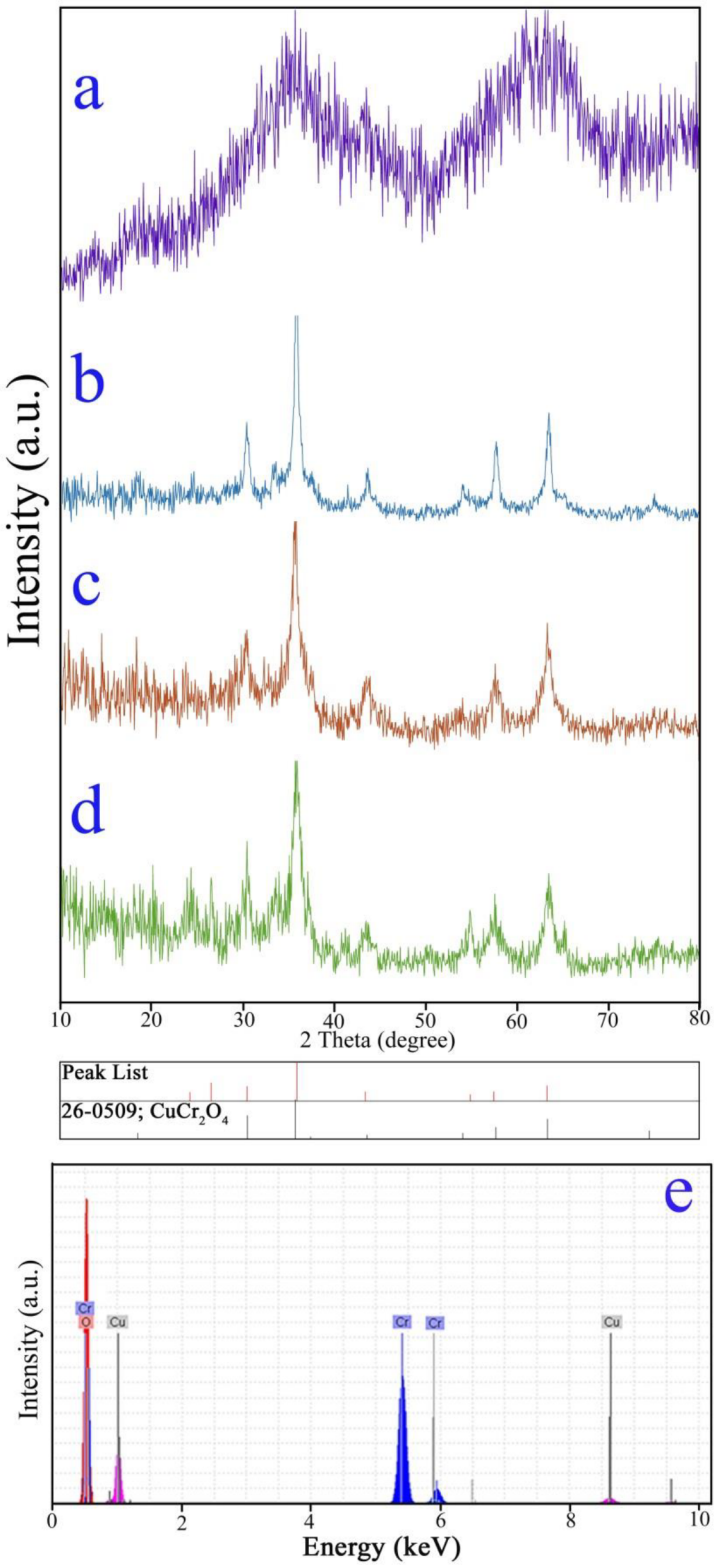
XRD patterns of the sample no. 1 after washing steps (a) and copper chromite samples prepared at 600°C (b), 500°C (c), 400°C (d) and EDS pattern (e) of the copper chromite nanostructures (sample no. 3).

To characterize the crystalline structure of the as-obtained samples (sample no. 1 after washing steps, and sample nos. 1–3 after calcination), XRD patterns were taken and illustrated in Fig 3A–3D. As seen in Fig 3A, the precipitate synthesized by the hydrothermal procedure (before calcination) seems amorphous. Fig 3B–3D exhibits XRD patterns of the samples prepared at 600, 500 and 400°C, respectively. All the diffraction peaks seen in Fig 3B–3D are well-matched to pure cubic CuCr_2_O_4_ (JCPDS card 26–0509). The mean crystallite size of the copper chromite samples prepared at 600, 500 and 400°C estimated by the Scherrer equation [2] are 22, 17 and 14 nm, respectively. So, pure cubic copper chromite with small mean crystallite size is prepared by calcination at 400°C.

In order to examine the chemical composition and purity level of the as-obtained copper chromite nanostructures (sample no. 3), EDS analysis was performed. The EDS spectrum of the sample no. 3 is illustrated in Fig 3E. The EDS spectrum indicates that this synthesized sample containing Cu, Cr and O elements. Therefore, the obtained FT-IR, XRD and EDS results demonstrate the high purity of the as-synthesized copper chromite nanostructures.

**Fig 4:**
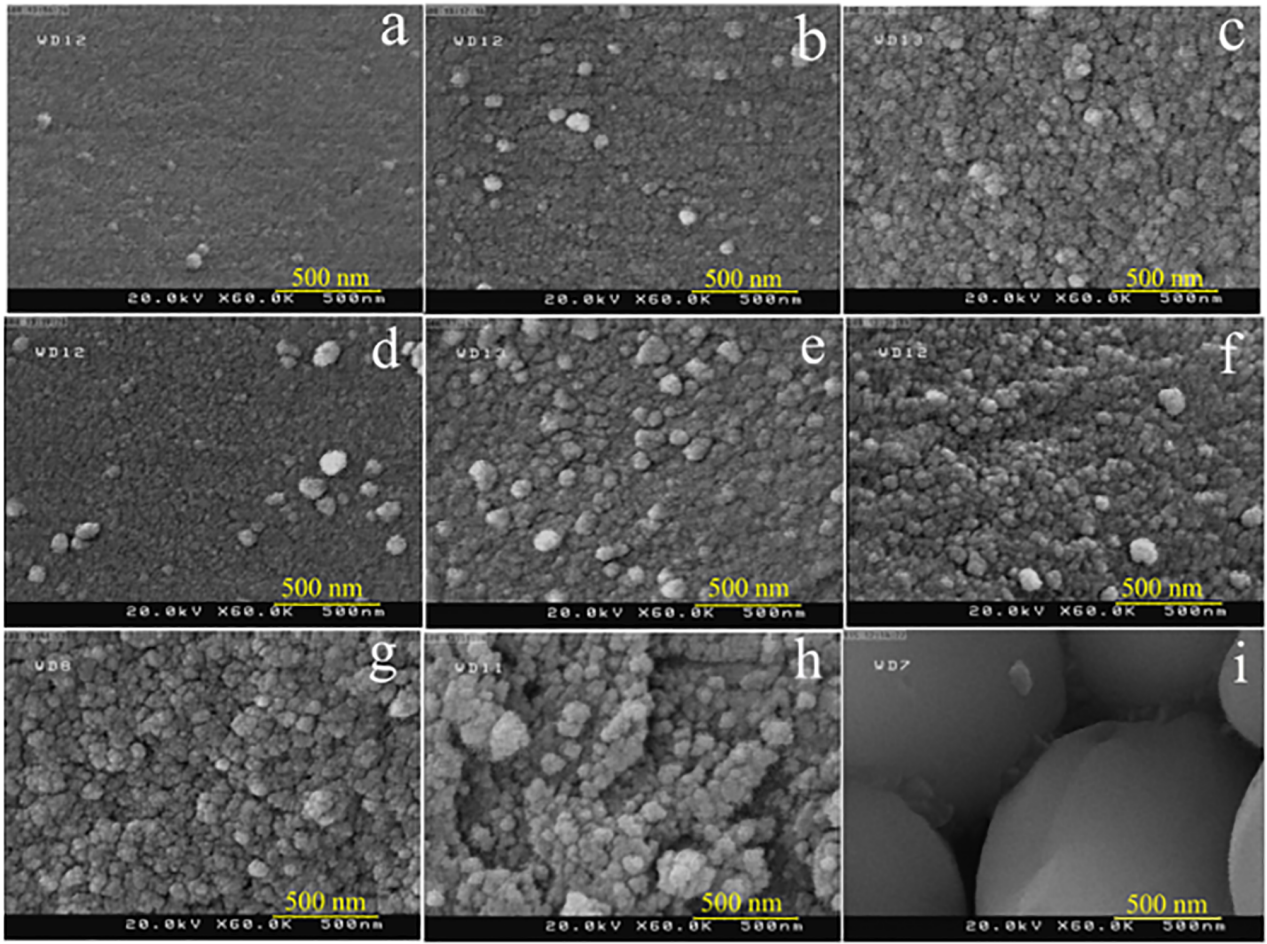
SEM images of the samples prepared at (a) 120°C, (b) 150°C and (c) 180°C for 6 h, at 120°C for (d) 12 and 18 h (e) and SEM images of the samples prepared in the absence of (f) CTAB, (g) PVP, SDS (h) and SEM image of sample no. 11 obtained from CuCl_2_.2H_2_O and CrCl_3_.6H_2_O in presence of NH_3_ as pH regulator via hydrothermal method (i).

As described before, in this study nanostructured copper chromite was synthesized through a novel facile hydrothermal reaction between [Cu(en)_2_(H_2_O)_2_]Cl_2_ and [Cr(en)_3_]Cl_3_.3H_2_O at low temperature. In this procedure, separated en from copper source and chromium source in the system has been applied as both pH regulator and capping agent. Preparation of copper chromite at 120°C for 6 h has been selected as a basic reaction in this research and the effects of the surfactant sort, reaction duration and temperature on the shape and size of the copper chromite has been examined by SEM technique. The reaction temperature influence on the shape and particle size of the copper chromite was studied (Fig 4A–4C). For this aim, the reactions were carried out at 120, 150 and 180°C (sample nos. 3–5). The SEM images illustrate that very uniform sphere-like nanostructures, less uniform sphere-like nanostructures and not uniform spherical nanostructures with large grain size are synthesized at 120, 150 and 180°C, respectively. It can be seen that the grain size becomes larger and the amount of uniform copper chromite nanostructures decreases by changing the temperature from 120 to 180°C, (Fig 4A–4C). It seems that when reaction temperature changed from 120 to 150 and 180°C, the nanoparticles with small grain size which were formed at 120°C agglomerated and fused to each other and therefore the grain size enhances. Results of SEM demonstrate that 120°C is the most desirable temperature for synthesizing nanostructures with uniform sphere-like shape (Fig 4A), therefore other reactions were carried out at this temperature.

Furthermore, the reaction duration influence on the size and shape of the copper chromite was examined. The reaction duration was changed to 12 and 18 h and the samples were prepared at 120°C (sample nos. 6 and 7). SEM images of the copper chromite sample nos. 6 and 7 obtained at 12 and 18 h were taken and illustrated in Fig 4D and 4E. As demonstrated in Fig 4D and 4E, when the reaction duration was prolonged (from 6 to 12 and 18h) not uniform sphere-like nanostructures and irregular spherical nanostructures with large grain size were formed, respectively. It seems that when reaction duration enhances, the enhancement in particle size occurs because of the Ostwald ripening process, and therefore the grain size becomes larger (Fig 4D and 4E). According to the SEM results, 6 h is the best reaction duration for uniform sphere-like copper chromite nanostructures with small particle size.

The effect of the surfactant type on the size and shape of the copper chromite was also investigated. SEM images of the copper chromite sample nos. 8, 9 and 10 synthesized by applying the CTAB, PVP and SDS were taken and seen in Fig 4F–4H. By applying the CTAB, PVP and SDS, not uniform spherical nanostructures, less uniform sphere-like nanostructures and irregular micro/nanostructures with large grain size are prepared, respectively (Fig 4F–4H). The obtained SEM results illustrated that applying these surfactant sorts not only is advantageous and desirable to prepare sample with a regular and uniform shape, but also leads to synthesize the not uniform and inhomogeneous samples. Perhaps owing to applying the [Cu(en)_2_(H_2_O)_2_]Cl_2_ and [Cr(en)_3_]Cl_3_.3H_2_O, there is no requirement to apply any other surfactant type. It seems that separated en ligand with the high steric hindrance effect from copper source and copper chromite in the reaction system can play a capping agent role to control the size and shape of the copper chromite.

…The influence of the Cu and Cr sources on the shape of copper chromite was evaluated. SEM image of the copper chromite sample no. 11 (as blank sample) prepared by applying the CuCl_2_.2H_2_O and CrCl_3_.6H_2_O in presence of NH_3_ as pH regulator was taken and seen in Fig 4I. The sample no. 11 reveals the bulk structures. The separated en with high steric hindrance from [Cu(en)_2_(H_2_O)_2_]Cl_2_ and [Cr(en)_3_]Cl_3_.3H_2_O in the system has been applied as both pH regulator and capping agent. It can be clearly seen that utilizing [Cu(en)_2_(H_2_O)_2_]Cl_2_ and [Cr(en)_3_]Cl_3_.3H_2_O as copper source and chromium source results uniform sphere-like nanostructures formation (Fig 4A). Thus, an excellence of applying [Cu(en)_2_(H_2_O)_2_]Cl_2_ and [Cr(en)_3_]Cl_3_.3H_2_O is that they lead to produce nanostructured copper chromite.

**Fig 5:**
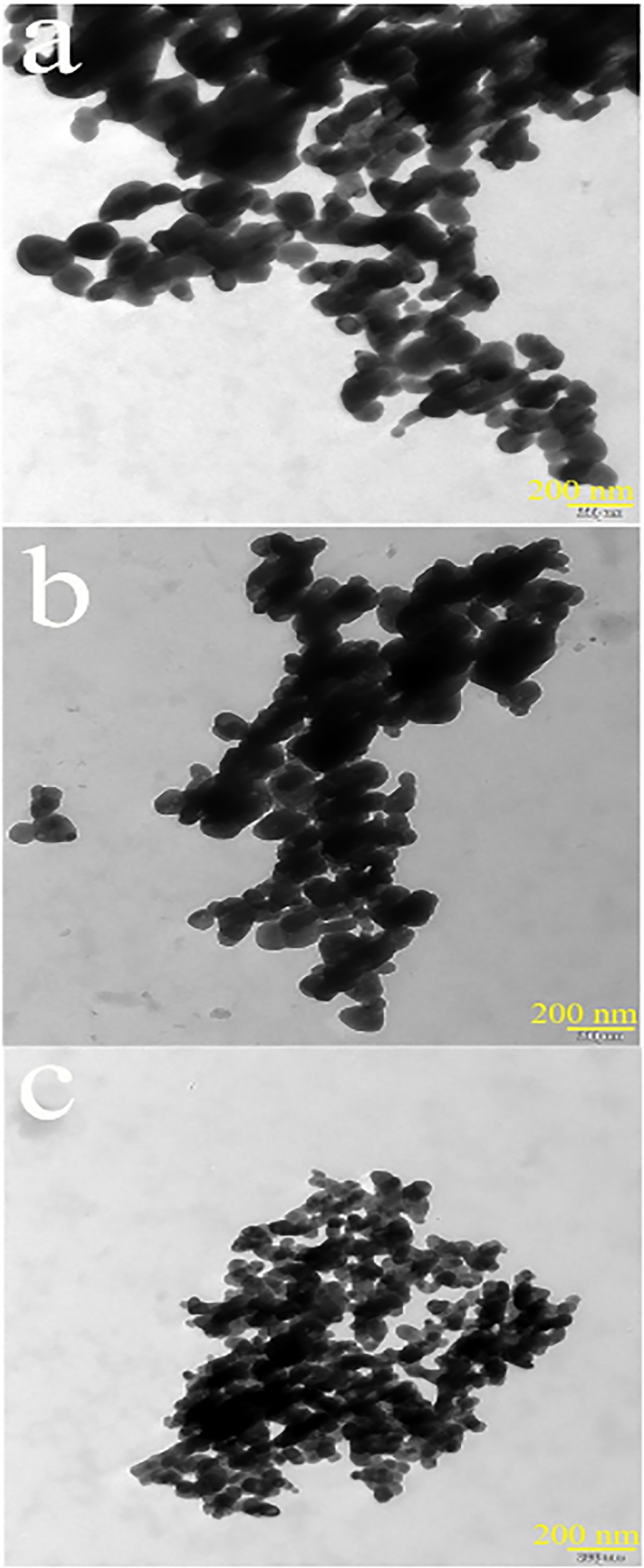
TEM images of the copper chromite nanostructures (sample no. 3).

To examine the detailed morphological characteristics of the as-obtained copper chromite sample in the optimum condition (sample no. 3) TEM images were taken. Fig 5A–5C exhibits TEM images of copper chromite sample prepare in the optimum condition (sample no. 3), illustrating quasi-spherical shape with size in the range of 22–55 nm.

**Fig 6:**
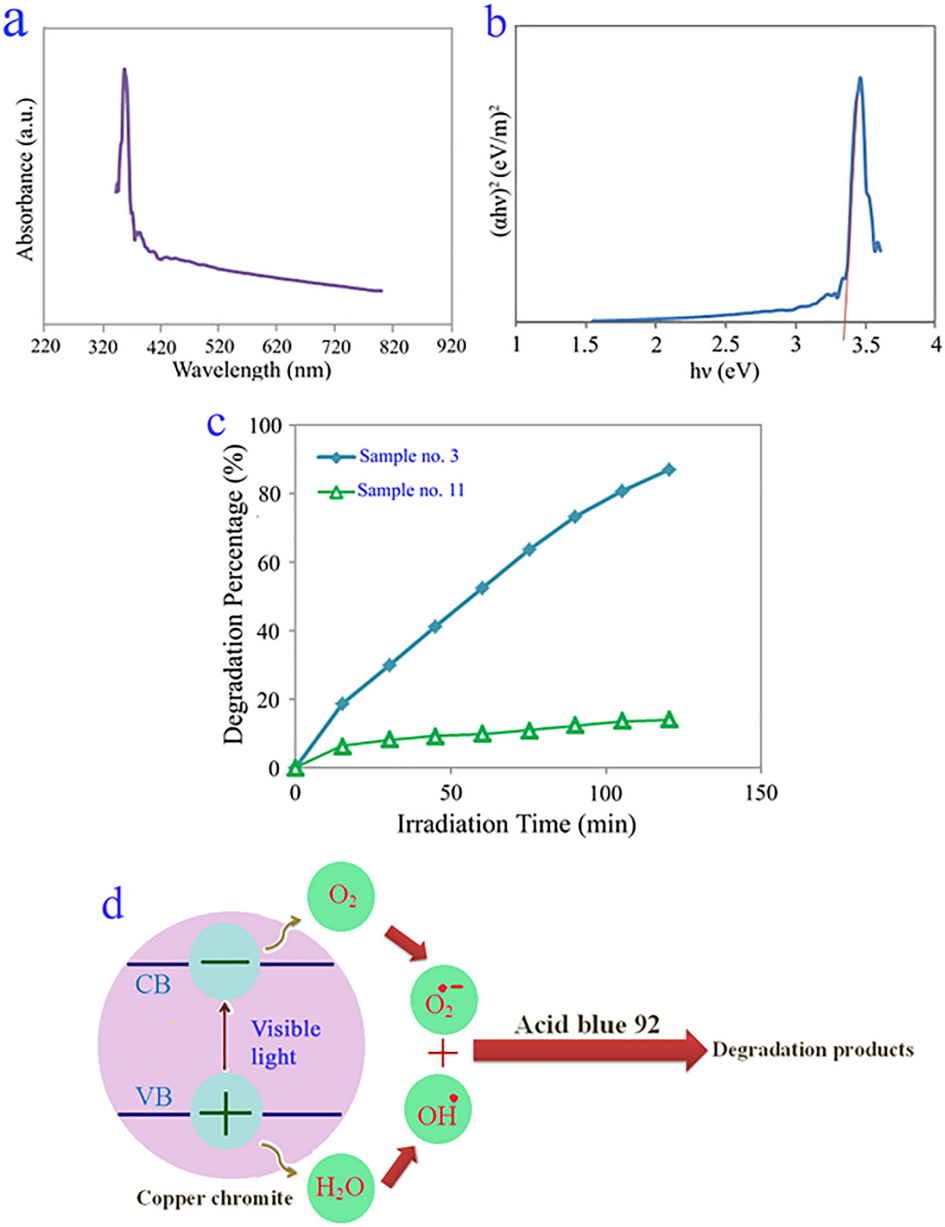
UV-vis diffuse reflectance spectrum (a), plot to determine the band gap (b) of the copper chromite nanostructures (sample no. 3), photocatalytic anionic dye (acid blue 92) degradation of the sample nos. 3 and 11 (c) and reaction mechanism of acid blue 92 photodegradation over copper chromite nanostructures under visible light illumination (d).

In order to detect the optical characteristics and band gap (*Eg*) of as-prepared copper chromite nanostructures, UV–vis diffuse reflectance spectroscopy was applied. Fig 6A illustrates the UV–vis diffuse reflectance spectrum of the copper chromite nanostructures (sample no. 3). The absorption peak at 359 nm is observed in Fig 6A. The *Eg* may be determined based on the absorption spectrum by applying Tauc’s equation [2]. The *Eg* of the copper chromite nanostructures can be calculated by extrapolating (*αhν*)^2^ against *hν* at (*αhν*)^2^ = 0 [1,4] (Fig 6B). The energy gap amount of the copper chromite determined to be 3.38 eV, which demonstrates a blue shift compared with the reported *Eg* value of copper chromite in prior documents [25] which this happened blue shift is corresponding to decrement in the particle size which cause alteration in particle energy levels and enhancement in the energy gap quantity. From the determined *Eg* quantity, it is found that the as-synthesized copper chromite nanostructures can be applied as the photocatalyst material.

The influence of shape on photocatalytic behavior was evaluated by monitoring photodegradation of acid blue 92 (anionic dye) as water pollutant over as-obtained sample nos. 3 (obtained by applying the [Cu(en)_2_(H_2_O)_2_]Cl_2_ and [Cr(en)_3_]Cl_3_.3H_2_O) and 11 (prepared by applying the CuCl_2_.2H_2_O and CrCl_3_.6H_2_O in presence of NH_3_ as pH regulator) with various morphology under visible light illumination. The obtained results are seen in Fig 6C. No acid blue 92 was practically broken down after 120 min without applying visible light or as-obtained copper chromite. This observation demonstrated that the contribution of self-degradation was insignificant. Applying photocatalytic calculations by Eq (1), the acid blue 92 degradation was about 87 and 14% over sample nos. 3 and 11, respectively, after 120 min illumination of visible light. This obtained result illustrates that uniform sphere-like nanostructures with small particle size (sample no. 3) have very good potential to be utilized as desirable and beneficial material for photocatalytic applications under visible light illumination. There are the diffusion, adsorption and reaction steps in the heterogeneous photocatalytic processes. It has been reported that the favorable and suitable distribution of the pore has beneficial and key impact on the diffusion of the reactants and products, and so influences on the photocatalytic behavior. It seems that the very good photocatalytic activity of the as-synthesized copper chromite nanostructures (sample no. 3) can be corresponding to favorable and suitable distribution of the pore, high hydroxyl quantity and very well separation rate of charge carriers [26] (Fig 6D).

In comparison to other investigations, illustrated in Table 2, our way is more cost-effective, facile, reliable and friendly to the environment. In this study, we introduced a novel simple hydrothermal procedure to prepare copper chromite nanostructures applying [Cu(en)_2_(H_2_O)_2_]Cl_2_ and [Cr(en)_3_]Cl_3_.3H_2_O in the presence of water as nontoxic solvent at low temperature. The novelty of this study compared to other works is that for the hydrothermal synthesis of the copper chromite, [Cu(en)_2_(H_2_O)_2_]Cl_2_ and [Cr(en)_3_]Cl_3_.3H_2_O were applied. In this new hydrothermal procedure, separated en from copper source and chromium source in the system has been employed as both pH regulator and capping agent. In other cases (Table 2), the pH regulator and surfactant as one of the starting materials has been added in the reaction system. Results of this investigation illustrate that the simple hydrothermal reaction between [Cu(en)_2_(H_2_O)_2_]Cl_2_ and [Cr(en)_3_]Cl_3_.3H_2_O leads to the synthesis of the copper chromite nanostructures with high purity, uniform sphere-like shape and small crystallite size at lowest temperature (Table 2).

**Table 2:** Characterization comparison of copper chromite nanostructures with other similar works.

Method	Precursors (condition)	Size (nm)		Morphology	Ref.
Hydrothermal route	[Cu(en)_2_(H_2_O)_2_]Cl_2_ and [Cr(en)_3_]Cl_3_.3H_2_O (needed heating at 120°C for 6 h and calcining at 400°C)	14 nm	Uniform sphere-like nanoparticles		This work
Hydrothermal method	CuCl_2_·2H_2_O, CrCl_3_·6H_2_O, NH_3_, CTAC, hydrazine (needed heating at 200°C for 18 h and calcining at 700°C)	35 nm	Less uniform sphere-like particles		[14]
Hydrothermal route	Cu(NO_3_)_3_·3H_2_O, Cr(NO_3_)_3_·9H_2_O, NaOH, (needed heating at 180°C for 11 h and calcining at 600°C)	18 nm.	Not uniform cubic-like structures		[7]
Hydrothermal method	CuSO_4_·5H_2_O, Cr_2_(SO_4_)_3_·12H_2_O, NH_4_OH, hydrazine hydrate, CTAB, (needed heating at 180°C for 18 h and calcining at 750°C)	38 nm	Less uniform sphere-like particles		[27]
Hydrothermal route	Cu(NO_3_)_2_·3H_2_O, Cr(NO_3_)_3_·9H_2_O, ammonia, CTAB, hydrazine (needed heating at 180°C for 28 h and calcining at 750°C)	38 nm	Less uniform sphere-like particles		[20]

## Conclusion

This work presents a novel simple hydrothermal way based on hydrothermal reaction between [Cu(en)_2_(H_2_O)_2_]Cl_2_ and [Cr(en)_3_]Cl_3_.3H_2_O for the preparation of copper chromite nanostructures at low temperature. The novelty of this research is that for the hydrothermal preparation of the copper chromite, [Cu(en)_2_(H_2_O)_2_]Cl_2_ and [Cr(en)_3_]Cl_3_.3H_2_O were employed. In this novel hydrothermal way, separated en from copper source and chromium source in the system has been applied as both pH regulator and capping agent. By alteration of the surfactant sort, reaction duration and temperature, we could synthesize copper chromite with different shapes and grain sizes. When as-synthesized copper chromite was applied as photocatalyst, the percentage of the acid blue 92 degradation was about 87 after 120 min illumination of visible light. This result suggests as-prepared copper chromite as interesting and desirable candidate for photocatalytic applications under visible light.
